# Near-infrared spectroscopy of low-transmittance samples by a high-power time-stretch spectrometer using an arrayed waveguide grating (AWG)

**DOI:** 10.1038/s41598-023-44359-1

**Published:** 2023-10-12

**Authors:** Hiroyuki Kawagoe, Hideyuki Sera, Junki Sahara, Shingo Akai, Katsuya Watanabe, Kazuki Shinoyama, Toshikazu Nagashima, Takuma Yokoyama, Aya Ikarashi, Go Yamada

**Affiliations:** New Technology Development Department, R&D Division, Business Creation Division, USHIO INC., 6409 Motoishikawa, Aoba, Yokohama, Kanagawa 225-0004 Japan

**Keywords:** Near-infrared spectroscopy, Fibre optics and optical communications

## Abstract

Although time-stretch spectroscopy is an emerging ultrafast spectroscopic technique, the applications in industrial fields have been limited due to the low output power caused by undesirable nonlinear effects occurred in a long optical fiber used for pulse chirping. Here, we developed a high-power time-stretch near infrared (NIR) spectrometer utilizing arrayed waveguide gratings (AWGs). The combination of AWGs and short optical fibers allowed large amounts of chromatic dispersion to be applied to broadband supercontinuum pulses without the power limitation imposed by employing the long optical fiber. With the proposed configuration, we achieved chirped pulses with the output power of 60 mW in the 900–1300 nm wavelength region, which is about 10 times higher than conventional time-stretch spectrometers using long optical fibers. With the developed spectrometer, the NIR absorption spectra of a standard material and liquid samples were observed with high accuracy and precision within sub-millisecond measurement time even with four orders of magnitude optical attenuation by a neutral density filter. We also confirmed the quantitative spectral analysis capability of the developed spectrometer for highly scattering samples of an oil emulsion. The qualitative comparison of the measurement precision between the developed spectrometer and the previous time-stretch spectrometer was also conducted.

## Introduction

Near-infrared (NIR) spectroscopy is an emerging technique for rapid and non-destructive quality assessment in various industrial fields. An NIR spectrum provides information about the molecular vibrations of the sample, which allows the monitoring of chemical properties such as moisture content, chemical constituents, and so on^1,2^. In addition, NIR light has the ability to penetrate deeper into the samples than IR light because the absorption of the combination and overtone bands appearing in the NIR wavelength region is much weaker than that of the fundamental absorption peaks in the IR region. This allows us to obtain chemical information inside or through a thick sample non-invasively. Recent developments in computational techniques such as chemometrics and machine learning, which enable quantitative spectral analysis, are also promoting practical applications in many fields, including food, agriculture, pharmaceuticals, and chemicals^3–6^. Total inspection in production is one of the attractive applications to fully exploit the unique nature of NIR spectroscopy, but it is still challenging mainly due to the slow spectral acquisition rate of conventional NIR spectrometers. Fourier Transform Infrared (FT-IR) spectrometers need the precise mechanical scanning of the optical path length of the interferometer within the system to record the interferogram, limiting the spectral acquisition rate between a few to tens of Hz. For example, to achieve the spectral resolution of 10 cm^−1^, the mirror in a Michelson interferometer has to be scanned 0.5 mm under a precise control of movement. Although diffraction-grating-based NIR polychrometers have a maximum spectral scan rate of a few kHz, the practical measurement rate is limited to several hundred Hz or lower. Because the throughput of polychromators is limited by the entrance slit and a low numerical aperture, the spectral averaging is required to obtain the NIR spectrum with a high signal-to-noise ratio (SNR).

In recent years, high-speed spectroscopic methods using chirped (temporally dispersed) optical pulses, called time-stretch spectroscopy or dispersive Fourier transform spectroscopy, have emerged as a promising alternative^7–9^. In these methods, the optical spectrum is converted from the temporal waveform of the chirped pulse measured with a single high-speed photodetector at a pulse repetition rate exceeding MHz frequency. Studies of instantaneous dynamics, including optical pulse evolution and gas mixtures, have been successfully demonstrated using time-stretch spectroscopy^10–12^. To apply this technique to high-speed NIR spectroscopy of industrial objects that are usually low-transmittance, it is essential to improve the output power of time-stretch spectrometers. Conventionally, most time-stretch spectrometers have employed a kilometer-long optical fiber for pulse chirping due to its simplicity and robustness^7–9^; however, the accessible optical power is limited by undesirable nonlinear effects in the long optical fiber. For example, the stimulated Raman effect and four-wave mixing limit the available output power at the desired wavelength because the excess energy above their nonlinear threshold is converted to other wavelengths^13,14^. To realize the highly sensitive measurement while avoiding the undesirable nonlinear effects, an advanced configuration using distributed Raman amplifiers has been demonstrated^15^; however, the system becomes somewhat complicated. Another approach based on free-space angular-chirp-enhanced delay (FACED) has recently been demonstrated^16^. However, the need for careful alignment of bulky optical components (a diffraction grating and large mirrors) may limit its feasibility in industrial applications.

In this study, we demonstrated NIR spectroscopy of low-transmittance, highly-scattering samples with a time-stretch spectrometer using arrayed waveguide grating (AWG) technology. The concept of the developed spectrometer was previously described in our patent^17^. AWG is an optical waveguide device commonly used in optical communications for spatially separating and combining light waves of multiple wavelengths^18,19^. The combination of AWGs and short optical fibers allows us to generate temporally dispersed pulse trains at different wavelengths while avoiding the power limitation caused by using long optical fibers. Our developed time-stretch spectrometer using AWGs achieved a high output power of 60 mW in the wavelength range of 900–1300 nm, which is about 10 times higher than that of conventional time-stretch spectrometers based on long fibers^11,12^. With the developed spectrometer, we performed transmission spectroscopy of opaque samples. The absorption spectra of low-transmittance samples were observed with high accuracy and precision in sub-millisecond measurement time. We also confirmed the quantitative spectral analysis capability of the developed spectrometer for highly scattering samples (oil emulsion).

## Results

### AWG-based high-power time-stretch spectrometer

**Figure 1:**
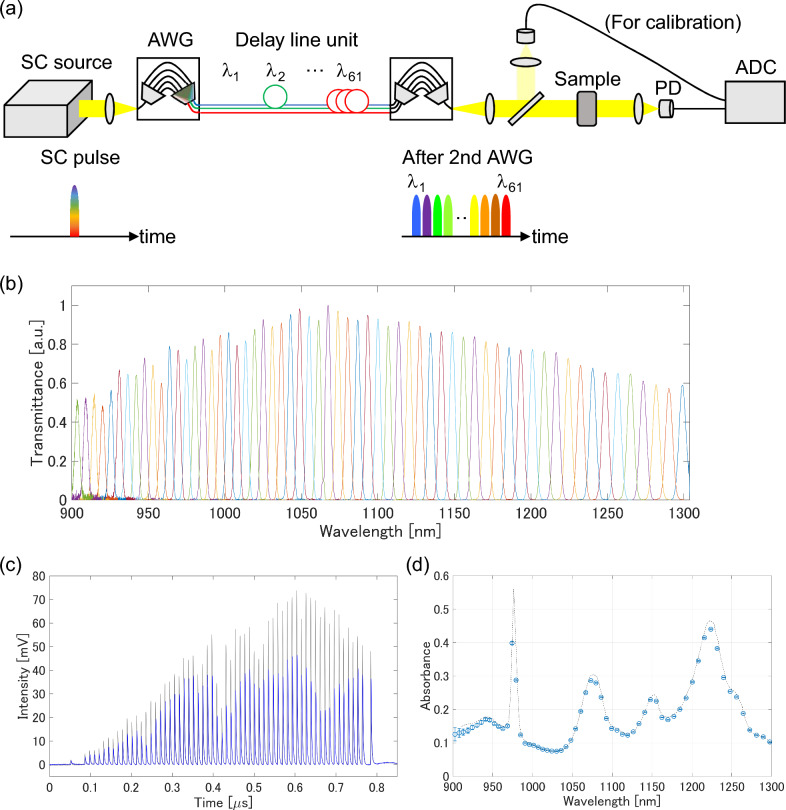
AWG-based high-power time-stretch spectroscopy. (**a**) Schematic of the developed spectrometer. The relationship between the optical spectrum and the pulse waveform is shown in the bottom row. *SC* supercontinuum, *AWG* arrayed waveguide grating, *PD* photodiode, *ADC* analog-to-digital converter. (**b**) Transmittance spectra of the AWG (61 output channels) in 900–1300 nm wavelength range. (**c**) Pulse waveforms measured without (gray) and with (blue) a standard reference material. (**d**) Measured absorption spectrum of the standard material (circles). The reference spectrum provided by NIST is also shown as dashed lines. The error bars represent the standard deviation of absorbance (± 1σ). The experimental conditions are given in the “Methods” section.

Figure 1a shows the schematic setup for time-stretch NIR spectroscopy with the spectrometer that we developed. A custom-made supercontinuum (SC) source covering the wavelength range of 900–1300 nm with a pulse repetition rate of 1.2 MHz was used as the light source. The SC pulse was split into sub-pulses of different wavelengths by the first AWG. The transmittance spectrum of the AWG is shown in Fig. 1b, indicating that the SC spectrum was split into 61 spectral channels with negligible spectral crosstalk between each other. To introduce a temporal delay for the sub pulses, the output ports of the first AWG were connected to a delay line unit consisting of optical fibers of different lengths. This configuration allowed us to achieve a large temporal delay for a broadband spectrum with short length optical fibers. In this study, we increased the fiber length by 2.35 m at each output channel of the AWG, resulting in 11.5 ns delay between each adjacent sub-pulse. Finally, the chirped pulse train was obtained by recombining the 61 sub pulses into a single output channel with the second AWG. By eliminating the need for a km-long optical fiber, a high output power of 60 mW was achieved in the 900–1300 nm wavelength region. Full details of the developed spectrometer are given in the “Methods” section.

We characterized the measurement accuracy and precision of our spectrometer when measuring low-transmittance samples, which is our main target, with a standard reference material (2035b, NIST)^20^. Because the reference material is relatively highly transparent, a neutral density (ND) filter, which is commonly used to reduce the optical intensity with less wavelength dependence, was added in front of the reference material to imitate the measurement of low-transmittance samples. We used the ND filter with an optical density (OD) of 3.8, that is, the intensity of the sample beam decreased by about four orders of magnitude through the ND filter. Figure 1c shows the transmittance signal through the reference material and ND filter obtained at 0.83 ms (1000 times averaging was applied). The temporally dispersed sub pulses were observed within the period of SC pulses (0.83 μs) without cross-talk. The calculated absorption spectrum of the sample is shown in Fig. 1d with the measurement precision. The high accuracy of our spectrometer was confirmed by comparing the observed spectrum and the typical spectrum of the reference material shown in the certification sheet^20^. The high measurement precision was achieved for the wavelength above 950 nm (the coefficient of variation of < 2%); in contrast, the precision gradually degraded for shorter wavelengths mainly due to the low detection intensity caused by a low sensitivity of the InGaAs photo-detector we used. As shown here, our developed spectrometer achieved the high measurement accuracy and precision with sub-millisecond measurement time even though about four orders of magnitude optical attenuation by the ND filter.

### NIR spectroscopy of low-transmittance samples

**Figure 2:**
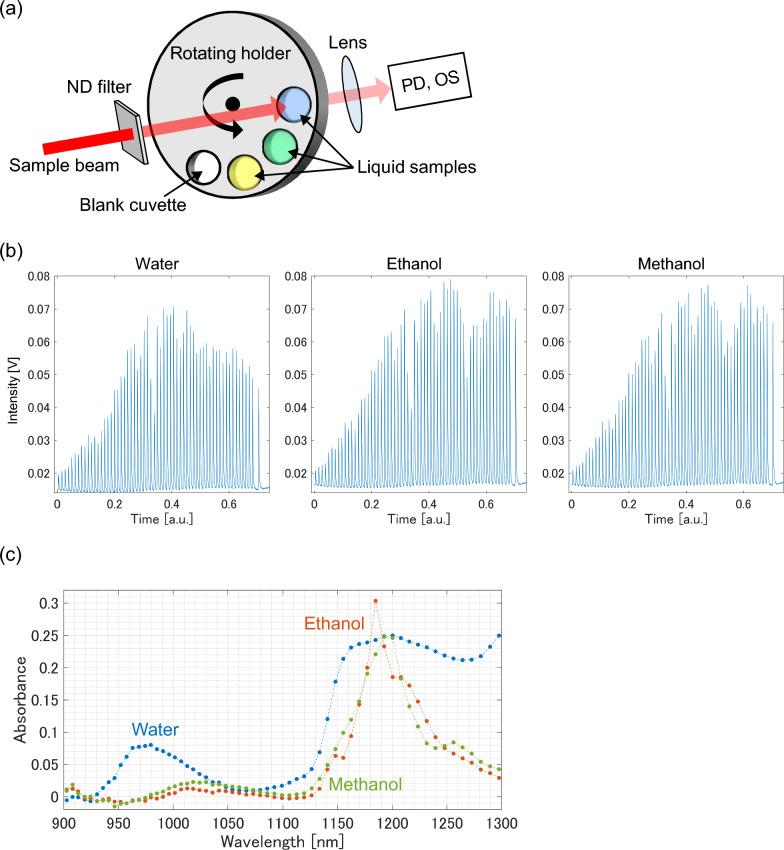
NIR spectroscopy of low-transmittance samples. (**a**) Schematic of the experimental setup. *PD* photodiode, *OS* oscilloscope. (**b**) Waveforms observed through liquid samples. We averaged 1000 successive waveforms for each sample extracted from the raw signal (Fig. S2). (**c**) Absorption spectrum of the samples measured by the developed spectrometer.

By using the developed spectrometer, we performed NIR spectroscopy of low-transmittance samples. As the proof of concept for the total inspection in industrial field, the absorption spectrum of the samples (liquids of water, ethanol, and methanol) moving fast was observed. Same as in the previous section, we added the ND filter (OD 3.8) in front of the liquid samples to imitate the measurement of low-transmittance samples. A schematic diagram and photograph of the experimental setup are shown in Fig. 2a and Supplementary Fig. S1, respectively. The transmittance signal through the samples was continuously recorded with an oscilloscope while rotating a sample holder (see Supplemental Movie 1). The raw transmittance signal is plotted in Supplementary Fig. S2. Note that, the liquid samples crossed the sample beam only once during the signal acquisition time shown in Fig. S2. To calculate the absorption spectrum, we averaged 1000 sequential sets of the chirped pulse trains for each sample, which corresponds to a measurement rate of 1.2 kHz. The averaged waveforms are shown in Fig. 2b. The wavelength-dependent intensity difference among the samples was clearly observed with a high SNR. The calculated absorption spectra of the samples (Fig. 2c) were in good agreement with the reference spectra obtained with a commercially available monochrometer (Supplementary Fig. S3), indicating the high accuracy of our spectrometer. Note that, the commercial monochromator required much longer measurement time (~ 1 min) to observe the NIR spectrum of the liquid sample even without the ND filter. As shown here, the developed spectrometer enabled us to perform NIR spectroscopy for low-transmittance samples (liquids and the ND filter with an OD of 3.8) in sub-millisecond measurement time.

### NIR spectroscopy of highly scattering sample

We also demonstrated NIR spectroscopy of a highly scattering sample, which is typical of industrial materials. As a highly scattering sample, we selected aqueous dilutions of Intralipos^21–23^ (a soybean oil emulsion). To confirm whether quantitative spectral analysis was possible, we assessed the determination coefficient (R^2^) of the calibration model of the volume fraction developed from the transmittance spectra of the samples. The details of spectral measurement, pre-processing, and data analysis are described in the “Methods” section.

**Figure 3:**
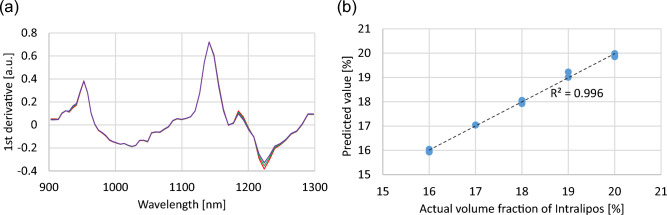
Results of NIR spectroscopy of highly scattering sample. (**a**) The first derivative of the transmittance spectra of Intralipos dilutions measured for 4.6 ms for each. The volume fractions of Intralipos were 20% (red), 18% (green), and 16% (blue). For each volume fraction, three samples were measured, and their spectra almost overlapped each other. (**b**) Plot of predicted and actual volume fractions of Intralipos in the sample dilutions.

Figure 3a shows the first derivative of the transmittance spectra of Intralipos dilutions measured with the developed spectrometer. The measurement time was 4.6 ms for each sample (5500 spectra were averaged for each). For the samples with different volume fractions of Intralipos, the difference in the spectral intensity around 1200 nm where the absorption peak of second overtone of C–H stretching appears^24^ can be seen clearly with a high SNR. Supplementary Fig. S4 shows the first loading component used to develop the calibration model, indicating the high contribution of the spectral components related with the lipid absorption (around 930 and 1210 nm)^24^ for development of the calibration model. The prediction results of the volume fraction of Intralipos dilutions using the developed model are plotted in Fig. 3b. A high R^2^ value of 0.996 was achieved, which also implies that our spectrometer can measure the spectral change according to the volume fraction of the sample at a high SNR. For comparison, we also performed the same analysis with NIR spectra measured with a commercially available FT-IR spectrometer. The R^2^ value was limited to 0.53 even with a longer measurement time (26 s/sample), probably due to the lower SNR of the measured spectra (see Supplementary Fig. S5). These results demonstrate the potential of our spectrometer for quantitative spectral analysis of highly scattering samples.

## Conclusion and discussion

In this study, we developed a time-stretch NIR spectrometer utilizing arrayed waveguide gratings (AWGs). The combination of AWGs and short optical fibers allowed us to introduce a large temporal dispersion to the broadband SC pulses. As a result, chirped pulse trains with a high output power of 60 mW were achieved in the 900–1300 nm wavelength range. The maximum spectral acquisition rate was 1.2 MHz. By using the developed spectrometer, we demonstrated NIR spectroscopy of low-transmittance and highly scattering samples. The absorption spectra of the low-transmittance samples (combination of a standard material or liquid and an ND filter with an OD of 3.8) were observed in sub-millisecond measurement time at high accuracy and precision. With highly scattering samples of soybean oil emulsion, we confirmed the potential of the developed spectrometer for quantitative spectral measurement at millisecond measurement time.

We expect that our spectrometer is suitable for industrial applications also in terms of the long-term reliability of the measurement due to the high stability against external disturbance. The spectroscopic characteristics including spectral accuracy and bandwidth in our spectrometer are solely determined by the AWG and they are almost insensitive to the optical alignment with optical fibers, ensuring the high stability to external vibrations. In contrast, in case of polychrometers those characteristics are varied by the relative position and angle between a grating and linear sensor. Although it is well known that the optical waveguide including the AWG is susceptible to the ambient temperature^25^, the small foot print of the AWG enables us to control the device temperature easily with such as small peltier devices.

Differently from conventional time-stretch spectrometers, our developed spectrometer is specialized for highly-sensitive broadband absorption spectroscopy at the expense of spectral resolution. Most conventional time-stretch spectrometers have been developed for the analysis of dynamics of gas mixtures or optical pulses in which a high spectral resolution such as few tens of picometer is required. The high spectral resolution has been realized by the high-speed sampling of optical spectrum of an optical pulse continuously chirped with a long optical fiber. In contrast, our spectrometer records the intensity of spectrally-divided optical pulses by the AWG, that is, at each measurement channel (sampling point in spectrum) we measure the optical intensity integrating for multiple wavelength components whose bandwidth is determined by the AWG. In this work, we designed our AWG with the bandwidth of 6.6 nm for each output channel (see Fig. 1b), yielding the hundreds of times improvement of the detection sensitivity at each measurement channel compared with conventional time-stretch spectrometers. Thanks to the improvement of the detection sensitivity and output power, our developed spectrometer achieved the similar level of measurement precision to the conventional broadband time-stretch spectrometer reported in Ref.^11^ even with four orders of magnitude optical attenuation by a ND filter. The details of the comparison are shown in Supplementary Note 1. Note that the spectral resolution of several nanometers should be acceptable for many industrial applications of NIR spectroscopy, because the absorption peaks of solid and liquid samples are much broader than that of gases.

The time-stretch spectrometer based on FACED geometry^16^ is also one of the possible systems for highly-sensitive and high-speed NIR spectroscopy. Since it uses only free-space optics (grating and mirrors), higher output power would be achieved than our spectrometer using the AWG and optical fibers. While it was used in mid-infrared wavelength region in the previous report, the operation in NIR region would be readily achieved because of the less wavelength dependence of the optical components used. However, its applications in industrial fields may be challenging due to the high susceptibility to the alignment of optical components in the system. The spectrum and amount of dispersion of chirped pulses are largely varied by slight changes in the angle and distance of two mirrors^26^.

Our spectrometer can be applied to not only broadband NIR spectroscopy, as shown in this manuscript, but also various spectroscopic measurements with modification of the optical design of the AWG device. Firstly, the operation wavelength range can be broadly changed from visible to short-wavelength infrared (SWIR). The wideband operation capability of optical waveguides using silicon nitride (Si_3_N_4_) has been successfully demonstrated in many reports^27–29^. For example to extend the wavelength range to 1700 nm where the strong absorption peaks of C–H and S–H appear, the analysis of organic materials including plastics and resins can be realized^30^. The recent development of SC sources with an extended spectrum in the visible to mid-IR wavelength range also encourages the extent of applications in broader wavelength region^31,32^. Secondly, high-resolution spectroscopy is also possible since AWGs were originally developed for high-density wavelength division multiplexing (WDM) optical communication^18,33^. Thanks to the higher output power derived from the proposed configuration, highly-sensitive monitoring of such as gases would be realized. However, one has to be careful that the improvement factor of the sensitivity will be somewhat limited because the narrower spectral width of the output channel of the AWG decreases the detection sensitivity of the transmittance signal at each measurement channel in spectrum. Lastly, even higher spectral acquisition rates can be achieved by reducing the number of spectral output channels of the AWG. By reducing the number of output channels, we can increase the pulse repetition rate of the SC source without cross-talk between temporally dispersed pulse trains. This configuration is still useful for some applications where it is necessary to monitor specific spectral bands.

## Methods

### AWG-based time-stretch spectrometer

A custom-made SC source (NKT Photonics) was used as the light source. In this work, we selected the wavelength range of 900–1300 nm for NIR spectroscopy because of the high spectral intensity of the SC source in the wavelength region. The other wavelength components were filtered out at the exit of the SC source by using long- and short-pass filters. The pulse duration was ~ 500 ps and the pulse repetition rate was 1.2 MHz. The SC pulse was coupled into the first AWG using an achromatic lens. In this study, we optimized the AWG design for broadband NIR spectroscopy in the 900–1300 nm wavelength region. The footprint of our AWG was 22 mm × 25 mm. The AWG split the SC pulse into 61 sub-pulses with center wavelengths spaced every ~ 56 cm^−1^ (~ 6.6 nm in wavelength). The peak transmittance of the 61 output channels of the AWG was 30–50%, mainly limited by the coupling loss of the SC pulse. The 1/e^2^ bandwidth of each output channel was ~ 6.6 nm. Although the AWG had three additional output channels below 900 nm, we did not use these channels for spectroscopic measurements. The sub-pulses were then transferred to the delay line unit consisting of 64 single-mode fibers to introduce a temporal delay between them. The fiber lengths ranged from 1 to 150 m, with a length increase of ~ 2.35 m, corresponding to a temporal delay of 11.5 ns applied to sub pulses at adjacent wavelengths. We determined the amount of temporal delay to avoid overlap between pulses in the time domain, taking into account the response time of our photodetector used in the spectroscopy. Finally, the temporally delayed pulses at different wavelengths were recombined into a single channel using the second AWG. The intensity of the recombined beam was ~ 60 mW. The total throughput of our spectrometer was about 7% at this moment, which can be further improved by increasing the coupling efficiency between the SC pulses and the AWG.

For absorption spectroscopy, the output beam from the second AWG was collimated to a diameter of 2.8 mm. The transmission signal through the sample was measured with a custom-made pin InGaAs photodiode (3 GHz bandwidth) and recorded using a high-speed oscilloscope (DSOS804A, Keysight) or digitiser (ADQ7DC, Teledyne SP Devices). A small portion of the sample beam was picked up via an optical window and recorded simultaneously with the sample beam to compensate for the spectral intensity fluctuation of the SC source. The transmittance spectrum of the sample was obtained from the area of each pulse signal after baseline correction using a polynomial fit. The absorption spectrum was calculated by dividing the transmittance spectra observed with and without the sample.

### Characterization of our spectrometer using a standard reference material

We measured the transmittance signal through the standard reference material (2035b, NIST^20^) and a ND filter with OD of 3.8 (Thorlabs). The OD of the ND filter slightly depended on the wavelength, and it varied from 4.0 to 3.7 in 900–1300 nm wavelength region. The signal was recorded with the oscilloscope (2.5 GHz bandwidth and 5 GHz sampling rate). To improve the SNR of the signal we averaged 1000 signals, corresponding to the measurement time of 0.83 ms. For the calculation of the absorption spectrum, the transmittance signal of only the ND filter was also measured as a blank signal. Therefore, the optical loss of the ND filter is canceled in the resultant absorption spectrum (Fig. 1d). For the evaluation of the measurement precision, we repeated the spectral measurement 50 times and calculated the standard deviation of the absorbance and its coefficient of variation.

### NIR spectroscopy of low-transmittance samples

The sample beam from the developed spectrometer passed through an ND filter (OD 3.8) and liquid samples (water, ethanol and methanol). The liquid samples were contained in glass cuvettes with a 5 mm path length (type 32/Q/5, Starna) and placed on a rotating plate. The radius of the plate was 180 mm, and there were 6.5 mm-diameter holes every ~ 19 mm around the circumference. The sample cuvettes were placed at every second well, and wells without samples were blocked. An empty cuvette was also provided to serve as a reference for calculating the absorbance of the samples. The plate was rotated at 160 rpm, corresponding to a sample moving speed of ~ 3 m/s. The sample beam was made to pass through the liquid sample for 2–3 ms, during which > 1000 spectra were observed at the 1.2 MHz pulse repetition rate of the SC source. The transmittance signal was recorded using an oscilloscope with a bandwidth of 2.5 GHz and a sampling rate of 5 GHz. A single non-averaged acquisition was performed after the rotational speed of the holder had stabilized at 160 rpm. To calculate the absorption spectrum of the samples, the signals generated by 1000 SC pulses were extracted from the raw signal and averaged for each sample to improve the signal-to-noise ratio. In the absorption spectrum (Fig. 2c), the optical loss by the ND filter is canceled same as the demonstration with the standard material.

To verify the accuracy of our spectrometer, the absorbance spectrum of the samples was also measured using a commercially available monochromator (V-7200, JSACO). A 10 mm path length cell (104-10-40, Hellma) was used. The measurement took ~ 1 min for each sample with a bandwidth of 2 nm and a recording pitch of 1 nm. The resulting absorbance was divided by two to estimate the absorbance for 5 mm path length (same path length as the measurement with our spectrometer). According to the specification sheet, the measurement precision of the monochromator is 0.00028 Abs.

### NIR spectroscopy of highly scattering samples

We prepared aqueous dilutions of Intralipos 20% (Otsuka Pharmaceutical) so that the volume fractions of Intralipos were 16, 17, 18, 19 and 20%. We prepared three samples for each of 16, 18, and 20%, and two samples for each of 17 and 19%. We measured the transmittance spectra of the dilutions with a 5 mm path length cuvette using the developed spectrometer and a commercially available FT-IR spectrometer (MPA, Bruker). For the measurement with the developed spectrometer, the illumination power on the sample was 60 mW, and the transmitted spectrum was recorded with a digitizer. We applied 5500 times spectral averaging to improve the SNR of the spectrum (measurement time was 4.6 ms for each sample). For the measurement with the FT-IR spectrometer, the resolution was set to 60 cm^−1^, and 128 spectra were averaged (measurement time was 26 s for each sample). For each system, we obtained 13 transmitted spectra (three spectra for each of 16, 18, and 20%, and two spectra for each of 17 and 19%) to develop the calibration model.

Spectral preprocessing and multivariate data analysis were performed using VEKTOR DIREKTOR (KAX Group). For spectral preprocessing, we applied standard normal variate (SNV)^34^ and Savitzky–Golay differentiation filter to develop the calibration model with high prediction accuracy. SNV transformation corrected variations in the baseline level of the spectrum caused by the scattering in the sample and scatters with different particle sizes. Savitzky–Golay differentiation filter reduced the noise in the spectrum and improved the separation of the absorption peaks overlap each other. We used a polynomial order and frame length of 2 and 3, respectively. We chose a differentiation order of 1 since higher differentiation orders can increase the random noise that can affect prediction accuracy although it improves the separation of the peaks overlap each other. Then, for each spectrometer, the calibration model for prediction of the volume fraction of Intralipos was developed with the 13 preprocessed spectra. We used the partial least squares (PLS) regression that provides a highly reliable prediction result by circumventing the multicollinearity problem of the NIR spectrum^35^. The accuracy of the developed model was validated using the leave-one-out cross-validation (LOOCV) method. The number of loading components used to develop the calibration model was chosen so that the mean squared error (MSE) between the predicted and objective variables became minimum. As a result, we used the only first and five components for the data sets obtained with the developed and FT-IR spectrometers, respectively (see Supplementary Figs. S4 and S5).

### Supplementary Information

Supplementary Information.

Supplementary Movie 1.

## Data Availability

The datasets used and/or analyzed during the current study are available from the corresponding author on reasonable request.
