# Supplementary material for: Near-infrared spectroscopy of low-transmittance samples by a high-power time-stretch spectrometer using an arrayed waveguide grating (AWG)
Source: Sci Rep. 2023 Oct 12;13:17261. doi: 10.1038/s41598-023-44359-1 (PMC10570277; doi:10.1038/s41598-023-44359-1)
Supplement: Supplementary file 1 — Supplementary Information. [file 41598_2023_44359_MOESM1_ESM.pdf]

Supplementary Information:

Near-infrared spectroscopy of low-transmittance samples by a high-power time-stretch spectrometer using an arrayed waveguide grating (AWG)

Hiroyuki Kawagoe, Hideyuki Sera, Junki Sahara, Shingo Akai, Katsuya Watanabe, Kazuki Shinoyama, Toshikazu Nagashima, Takuma Yokoyama, Aya Ikarashi, and Go Yamada.

Supplementary Note 1. Comparison of the measurement precision between our developed spectrometer and previous broadband time-stretch spectrometer.

Supplementary Figure S1. Photograph of the experimental setup for NIR spectroscopy of low-transmittance samples. An ND filter (OD 3.8) was inserted between the output of the spectrometer and liquid samples (not shown in this photograph).

Supplementary Figure S2. (a) Raw waveform observed with liquid samples mounted on a rotating holder. (b-d) Magnified view of (a) for (b) water, (c) ethanol, and (d) methanol.

Supplementary Figure S3. Absorption spectra of the liquid samples measured with a standard NIR monochromator.

Supplementary Figure S4. (a) The first loading spectrum obtained with multivariable data analysis for measurement of Intralipos performed by the developed spectrometer. The second to fifth loading spectra were almost entirely noise without significant peaks. (b) The mean squared error (MSE) between the predicted and objective variables for different number of loading components used to develop the calibration model.

Supplementary Figure S5. Results of multivariable data analysis for Intralipos measurement with a commercially available FT-IR. (a) Transmittance spectra of dilutions of Intralipos. The volume fractions of Intralipos in the dilutions were 20% (red), 18% (green), and 16% (blue). For each volume fraction, three samples were measured, shown as solid, dashed,

and long dashed dotted lines, respectively. (b) Loading spectra (first to fifth) obtained with the multivariable data analysis. (c) Plot of predicted and actual volume fractions of Intralipos in the sample dilutions. For the prediction, we used the five loading spectra above. (d) The MSE between the predicted and objective variables for different number of loading components used to develop the calibration model.

(Attached as a separate file) Supplementary Video 1. Movie of NIR spectroscopy for low-transmittance samples with a rotating sample plate. A red laser light was introduced collinearly with the NIR beam for clarity.

Supplementary Note 1. Comparison of the measurement precision between our developed spectrometer and previous broadband time-stretch spectrometer

We compare the measurement precision of our developed spectrometer with the previous broadband time-stretch spectrometer referenced as 11 in the main manuscript. We confirmed that our developed spectrometer achieved the similar level of precision to the previous one even with about four orders of magnitude optical attenuation.

To explain the conclusion above, we estimate the amount of transmittance light detected at each measurement channel (sampling point in spectrum). In the reference 11, the absorption spectrum of a gas sample was measured by the time-stretch spectrometer employing a SC source covering 1100 to 1700 nm wavelength and long optical fiber for pulse chirping. The SC pulses with the output power of 4.5 mW was introduced to the gas sample. Because of the repetition rate of the SC pulses of 1.1 MHz, the energy spectral density was calculated to ~7.5 pJ/nm for a SC pulse assuming the flat optical spectrum. The absorption spectrum was recorded by a high-speed oscilloscope with 20 GHz sampling rate, resulting in the sampling interval of ~20 pm (the spectral resolution was ~40 pm). According to the experimental condition above, the optical energy detected at a single measurement channel should be ~0.13 pJ for a SC pulse without an absorption sample. In our spectrometer, the intensity of the optical pulse with the spectral width determined by the AWG was measured at each measurement channel as mentioned in the main text. The pulse energy of each sub-pulse divided by the AWG was calculated to ~819 pJ (a SC pulse with 50 nJ was divided into 61 sub-pulses at different wavelengths by the AWG). Therefore, the optical energy detected at a single measurement channel was also ~819 pJ for a SC pulse without an absorption sample, which is ~6300 times higher than the previous one.

Then, we compared the experimental results of precision between the two systems. In the ref 11, the relative standard deviation of the absorption measurement was reported to ~50% without spectral averaging for the gas sample whose absorbance is ~0.3 (see Fig. 5 in the ref 11). In our study, even with the ND filter (OD 3.8), we obtained the relative standard deviation (coefficient of variation) of few tens of percent for the standard material whose absorbance is 0.1-0.6 without averaging as shown in the figure below. The

observed precision was the similar level to the previous report. This result is qualitatively reasonable considering the difference in the amount of detected light estimated above.

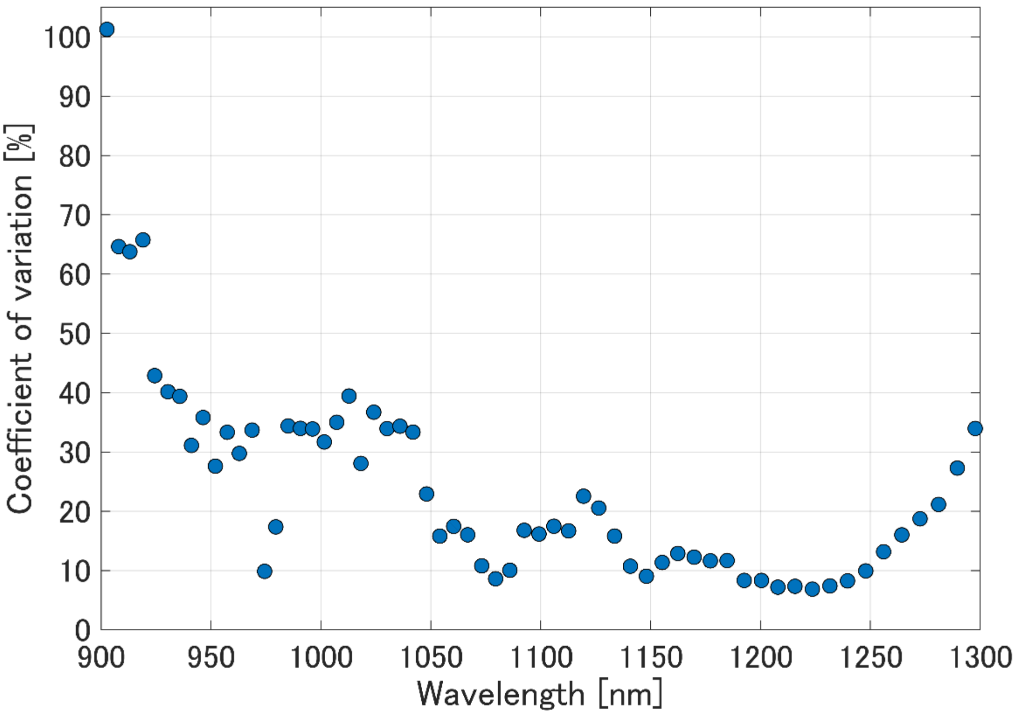

Figure. The coefficient of variation of absorbance measured with our spectrometer without spectral averaging for the standard material.

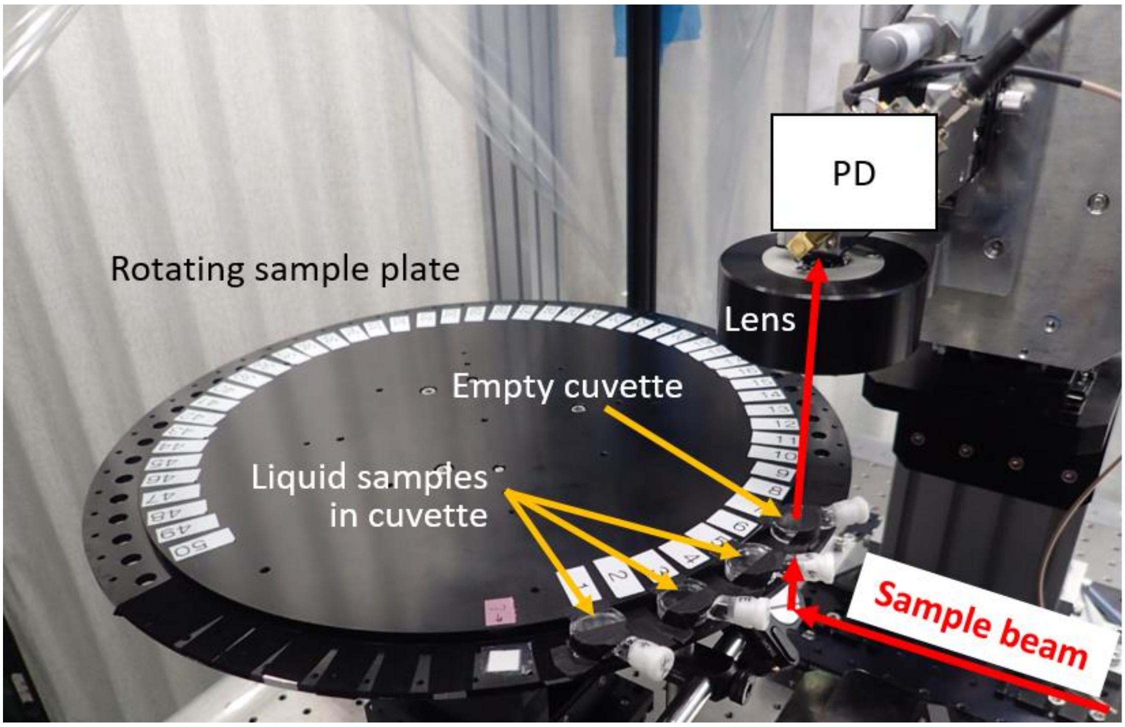

Supplementary Figure S1.

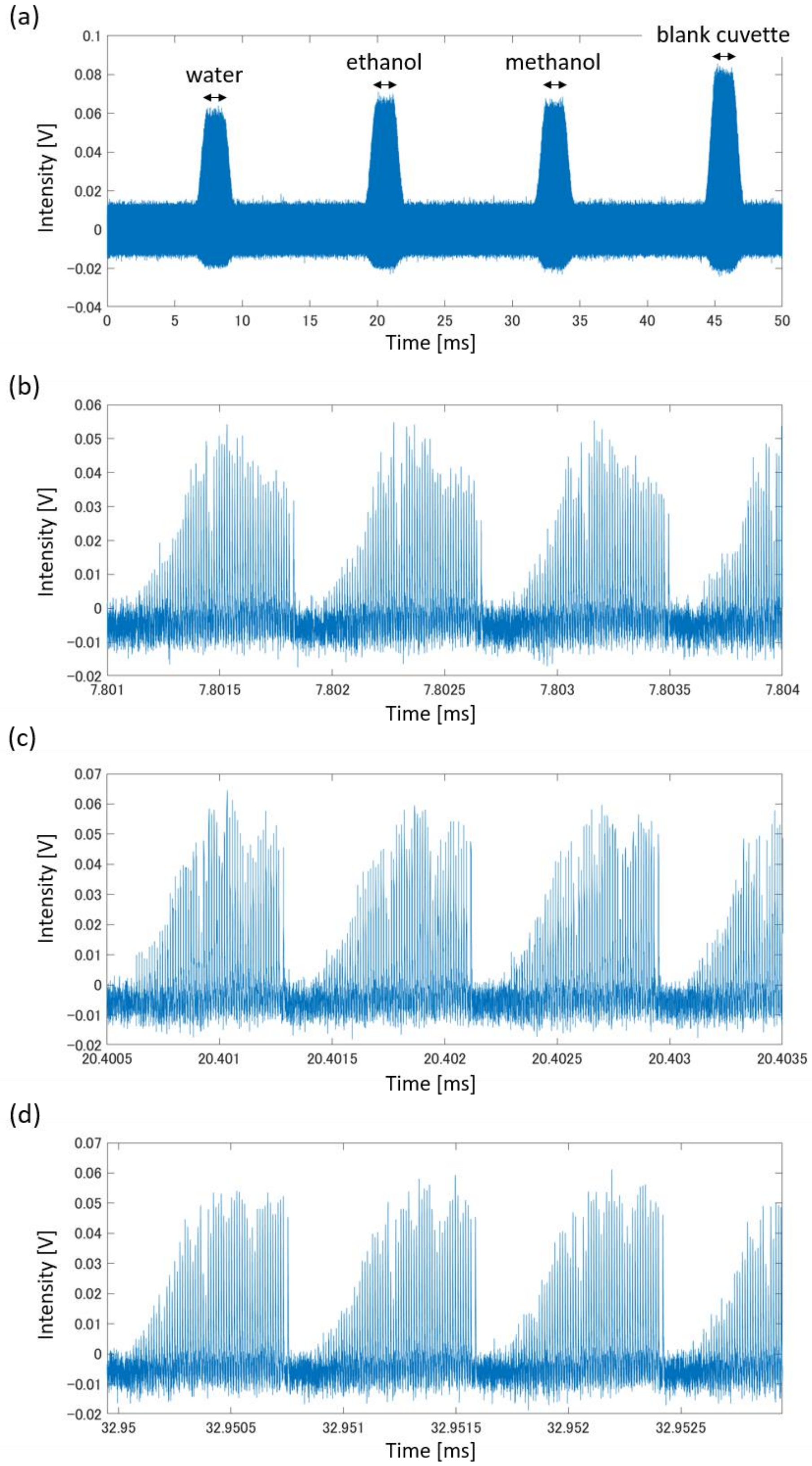

Supplementary Figure S2.

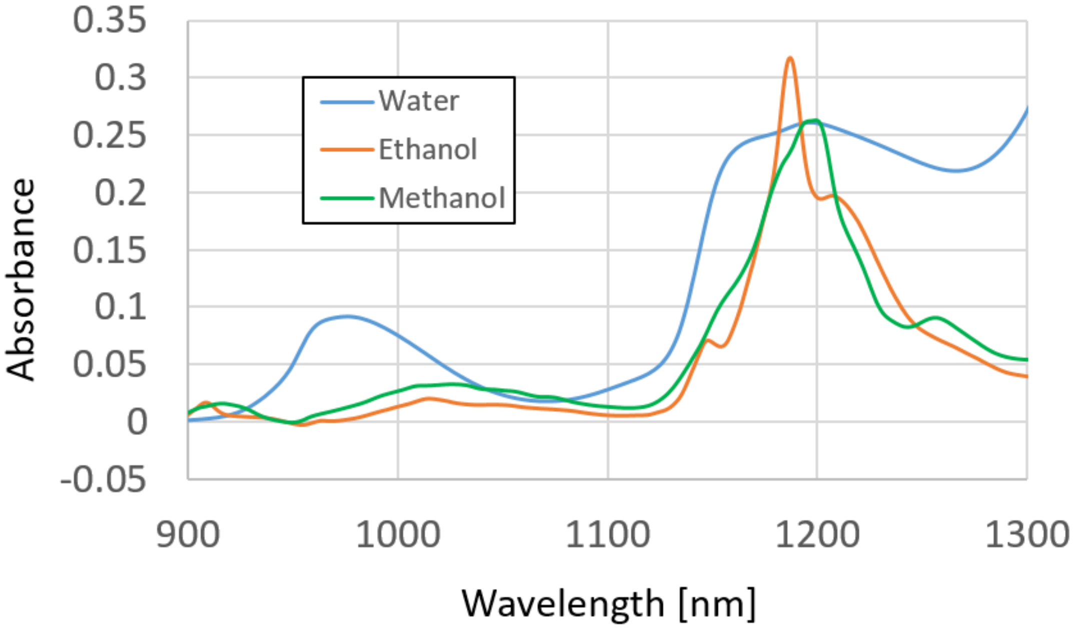

Supplementary Figure S3.

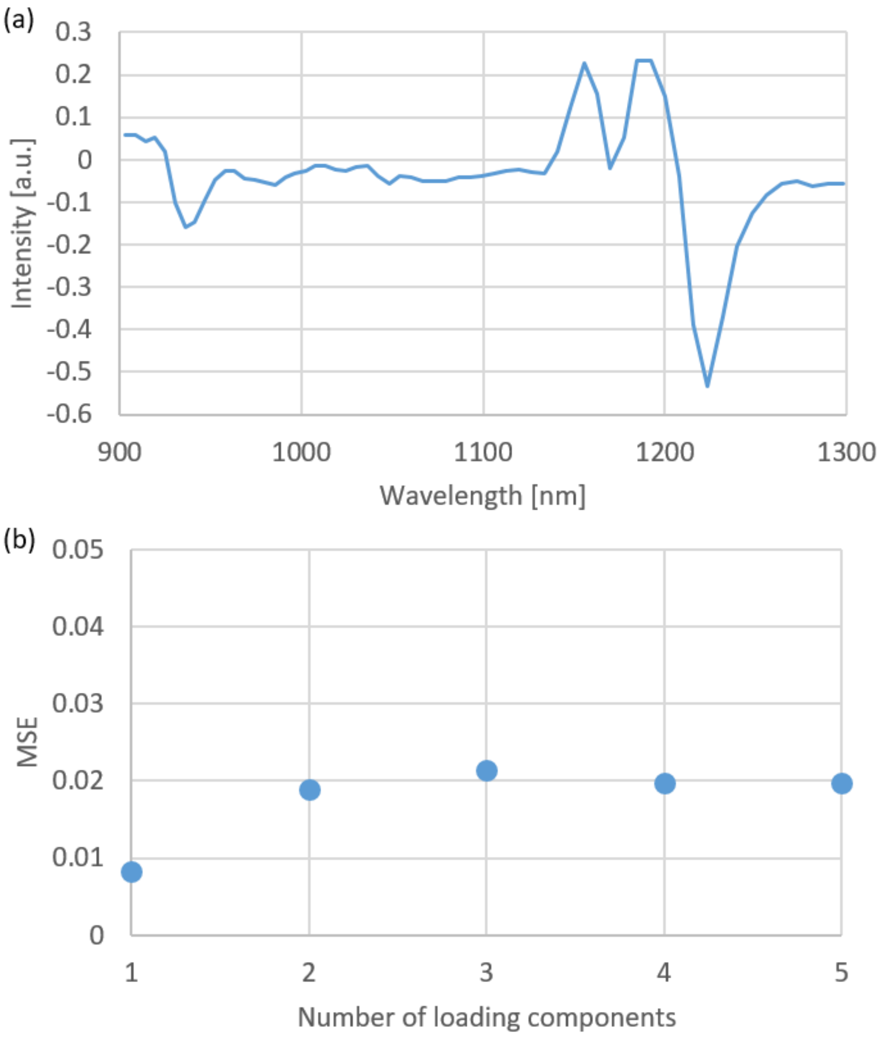

Supplementary Figure S4.

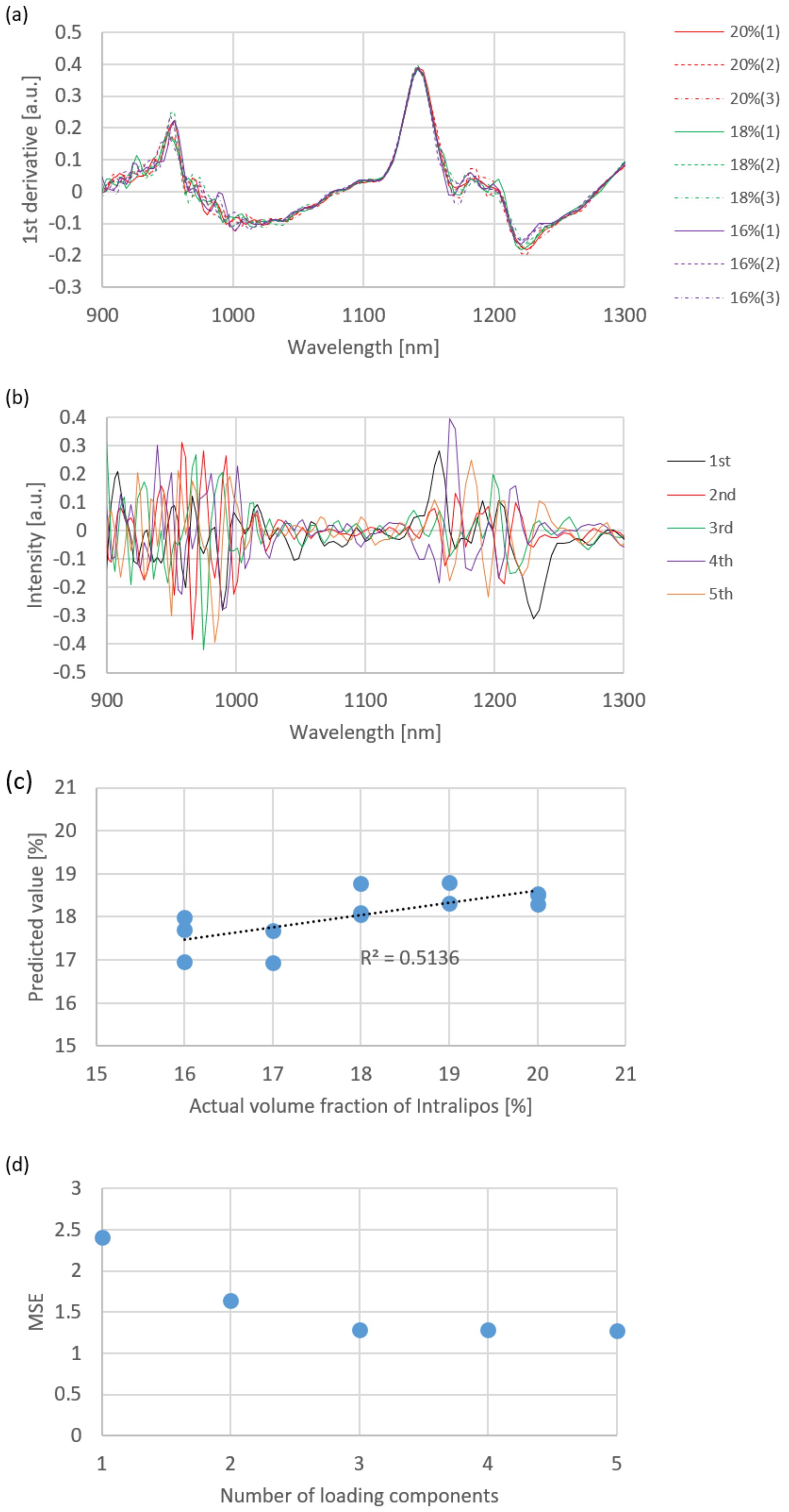

Supplementary Figure S5.
